# Effect of promethazine in cleft surgeries among Indian children

**DOI:** 10.6026/97320630019790

**Published:** 2023-06-30

**Authors:** Vedha Vivigdha A, Senthil Murugan P, Santhosh Kumar MP, Murugesan Krishnan, Sneha Alladi

**Affiliations:** 1 Department of Oral and Maxillofacial Surgery, Saveetha Institute of Medical and Technical Sciences (SIMATS), Saveetha University, Chennai, India

**Keywords:** Antihistamine, craniofacial surgery, promethazine, premedication, cleft palate

## Abstract

The use of antihistamine therapy in children for the management of upper respiratory tract infections remains a topic of debate. In this study, we focused on evaluating the effectiveness of promethazine (Phenergan), a first-generation H1 receptor antagonist and sedative, in addressing preoperative and intra-operative sequelae in cleft surgeries. A single-centered, parallel, randomized, double-blinded controlled clinical trial was conducted on 128 children aged 2 to 4 years undergoing cleft palate surgery under general anesthesia. The case group received Phenergan syrup orally twice a day for three days, while the control group received a placebo. Primary outcomes measured preoperative anxiety levels using a children's fear scale, while secondary outcomes assessed preoperative sleep quality and cough rate through objective scales. Intraoperative heart rate was monitored using an ECG connected to a monitor. The results demonstrated that the administration of promethazine resulted in a 34% reduction in anxiety levels, a 46% reduction in cold and cough, a 38% improvement in sleep score, and stable heart rates throughout the surgery compared to the control group. Based on these findings, promethazine is considered a safe premedication option for children undergoing cleft palate surgeries; given its benefits outweigh its adverse effects.

## Background:

Dental anxiety, characterized by tension, uneasiness, fear, and high autonomic activity, poses significant challenges for both patients and dental practitioners, leading to avoidance of dental care and associated complications [1,2]. This often causes fear in the patient's mind which may hinder the clinician's ability to appropriate treatment [3, 4]. The preoperative period is a horrible time for all patients, especially for young children undergoing surgery and Preoperative anxiety is a major and under-reported problem in children because of a lack of response and shortfall in standard methods of assessment [5]. Recent studies have indicated that up to 75% of children experience preoperative anxiety and fear in the preoperative holding area and during the induction of anesthesia [6]. This leads to increased postoperative delirium [7], increased postoperative pain [8], and an increased need for analgesia intra-operatively [8]. These negative behavioural changes linger even after one year after discharge from the hospital [9] and offset the child's future medical interactions thereby impeding the overall quality of life [10], therefore Intervention in preoperative anxiety is mandatory for the improvement of the child's welfare as well as the proper functioning of the healthcare system. Multitudinous pharmacological (e.g. sedatives, CNS depressants ) and non-pharmacological modalities (e.g. counselling, reinforcement program, parental presence, behavioural modification, white noise, acupuncture, etc.) have been successfully validated to reduce preoperative anxiety. Another common perplexity encountered in children due to prolonged hospital stays is nosocomial infections. India is a developing country the hospital-acquired infections like Nosocomial pneumonia and upper respiratory tract infections are common which leads to various complications and thereby deferring surgery [11]. Recently the outbreak of COVID virus [12], speculation remains about the transmission of the highly contagious virus through hospital stays children are susceptible to it [13]. This compulsory quarantine not only leads to economic crisis [14] but leads to Long-term acute panic, anxiety, repetitive habits, hoarding, hysteria, depression, and post-traumatic stress disorder (PTSD) in children [15]. Promethazine (PM) ((RS)-N,N-dimethyl-1-(10H-phenothiazine-10-yl)propan-2-amine hydrochloride) is a phenothiazine derivative. It is a first-generation H1 receptor antagonist, antihistamine, and antiemetic medication and can also have strong sedative effects [16]. It acts by blocking H1 receptor sites, thereby preventing the action of histamine on the cell. The antiemetic effect is related to dopaminergic receptor blockage in the chemoreceptor trigger zone (CTZ) of the medulla. Promethazine has strong anticholinergic effects, inhibiting acetylcholine responses mediated by muscarinic receptors thereby causing CNS depression. [17]. FDA has approved promethazine used to treat allergic conditions, nausea and vomiting, motion sickness, and as a sedative agent [18]. With these properties in mind, this study is done to check the effectiveness of promethazine as premedication for children undergoing cleft palate surgeries.

## Materials and Methods

This single-centered with randomized control clinical trial which was conducted in the Cleft and Craniofacial unit of Saveetha Dental College, Chennai, India. The study protocol was approved by the Saveetha Board of Ethical Committee (IHEC/SDC/2002/21/OSURG/586); a total of 128 patients undergoing isolated cleft palate surgeries were included in the study. We approached all patients aged between 2 to 4 years of age undergoing cleft palate surgery under general anesthesia (GA) between August 2021 and September 2022. The exclusion was patients undergoing surgery less than 2 years of age, lack of consent from patients or their parents, drug allergy, medically compromised children, syndromic children, or cognitive disorders, and children undergoing other surgeries than cleft lip and palate. All subjects were informed about the objectives and procedure of this study, and written consent was also obtained from each participant. Preoperative data were obtained from the subjects: Demographics (age, sex), Consanguinity of parents, and type of palatal defect (Kernahans classification). The participants were divided into 2 groups - the case group and the control group. The case group is 64 patients subjected to 2.5 ml of Phenergan syrup [orally] twice a day for 3 days before surgery is 64 patients.

## Procedure:

Patients were randomized 3 days before surgery by a trained nurse not involved in the perioperative patient care and using a computerized randomized sequence in a 1:1 ratio. At the time of randomization, the 128 patients were randomly allocated to receive phenergan 2.5 ml of Phenergan syrup twice a day orally for 3 days (case group). During the follow-up, the participants were monitored for possible side effects or drug discontinuation. Both the group patients were subjected to receive Budecort and Duolin nebulization OD morning on the day of surgery. Standard ASA monitoring (ECG, pulse oximetry, non-invasive blood pressure [BP], capnography, and temperature) was used for all patients. Anesthesia was induced with sevoflurane (6%-8% inspired in 100% oxygen) or intravenous propofol (2-5 mgkg1) and fentanyl (1-2 mgkg1) and it was maintained with sevoflurane 1.5% to 2.0% inspired in 100% oxygen. The airway was secured using an RAE tube. Spontaneous ventilation was maintained and supported when necessary to maintain ETCO2 between 30 and 35 mm Hg. All the cleft cases were operated using Bardach's palatoplasty and the duration of surgery was recorded. Precise details of each step like preoperative anxiety, intubation procedure, the duration of surgery, and intraoperative monitoring of heart rate medications given during surgery were documented.

## Ethical consideration:

The study purpose and procedures were explained to all parents of all enrolled children and written informed consent was obtained from each participant. The study was approved by the Ethical and Research Committee of the Saveetha Dental College.

## Outcome measures:

The primary outcomes were preoperative anxiety levels which were recorded by children's fear scale. The secondary outcomes include preoperative sleep quality and cough rate of children which is recorded by using sleep and cough objective scale respectively. The intraoperative heart rate is monitored with an ECG connected to a monitor.

## Children Fear Scale:

**Figure 1:**
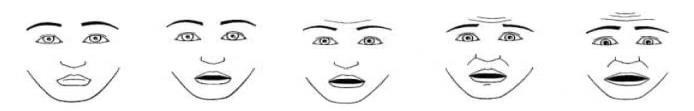
represents the Children's Fear Scale; these faces are showing different amounts of being scared. This face [point to the left-most face] is not scared at all, this face is a little bit more scared [point to the second face from left], a bit more scared [sweep finger along scale], and right up to the most scared possible [point to the last face on the right]. The scores are measured from 1 to 5 with 1 being normal and 5 being more anxious.

The children were observed in recovery 10 minutes before induction of anesthesia and the assessment is recorded by the same surgery trainee for all cases. The scale used was "The Children's Fear Scale" which was adapted from the Faces Anxiety Scale [19] to measure fear in children undergoing painful medical procedures (Figure 1).

## Preoperative complications

Parental assessment of the child's cough frequency and severity, sleep quality and post-tussive vomiting, and the parent's own sleep quality were measured and recorded after 24 hours of medication using an objective scale validated by Bhattacharya *et al*., given in Table 1 [20].

##  Heart rate

The heart rate is monitored using an ECG monitor connected using chest leads in all children undergoing cleft palate surgeries. The heart rate was noted for every 10 minutes from the beginning of surgery to the end time and the average was obtained.

## Results:

## Group statistics:

Children, where promethazine is given as premedication, were found to have a reduction in anxiety level by 34% and a 46 % reduction in cold and cough, an improvement in sleep score by 38%, and the heart rate was found to be stable throughout the surgery when compared to the control group and no aspiration difficulties were seen during intubation on patients taking Promethazine as premedication. Table 2 represents the Children where promethazine is given as premedication were found to have a reduction in anxiety level by 34.75% when compared to a control group with p value 0.044 (<0.05) which is found to be significant. Table 3 represents the Children where promethazine is given as premedication was found to have a 46.67% reduction in cold and cough when compared to the control group with a significant p-value of 0.001. Table 4 depicts the children where promethazine is given as premedication was found to have an improvement in sleep score by 38.67% with a p-value being significant 0.004. Table 5 represents the case group (N=64) had a mean heart rate of 105.2 beats per minute (bpm) during surgery, with a standard deviation of 7.6 bpm and the control group (N=64) had a mean heart rate of 113.7 bpm during surgery, with a standard deviation of 8.9 bpm The difference in mean heart rate between the case and control groups during surgery was statistically significant (p = 0.027), suggesting that the case group had a lower heart rate compared to the control group.

## Discussion:

Orofacial clefting is a worldwide deformity and contributes substantially to a quarter of the global burden of reconstructive surgical disease [21, 22]. Evidence shows that cleft lip and cleft palate are the most common craniofacial developmental abnormalities, affecting one in 700 live births [23]. The cleft palate children require surgical correction "Palatoplasty" which is the standard treatment for restoring palatal form and function [24]. They have detrimental effects on health and childhood development like malnutrition, feeding, and speech abnormalities which lead to psychological imbalance and social isolation of the child [21, 25]. Specifically, these children are observed to have lower self-esteem and struggle to interact socially [26]. Ramstad *et al*. found that anxiety and depression are twice prevalent in adults with cleft lip and palate compared with normal control [27]. They encounter diverse surgical and non-surgical treatments throughout their life [28], which leads to abnormal behavioral patterns before surgery. Another common problem faced by surgeons in cleft surgeries is that as these children are admitted to the hospital before surgery, they acquire upper respiratory tract infections. Nocturnal cough and sleep difficulty are the most commonly enumerated complaints among parents who bring their children to the hospital. In a study done by Shanuja *et al*. on children less than 5 years admitted to a Diarrheal Treatment Center in Bangladesh 84% of the study patients had nosocomial pneumonia [29]. Another study by Huxley *et al*. showed that 45% of healthy individuals aspirate during sleep, and aspiration is more frequent in patients with ailments requiring hospitalization [30]. Our study was conducted because a cough due to URI can be an exceedingly distressing symptom for the affected child, his parents, and the treating pediatrician. It is also responsible for school absenteeism, prompting parents to seek medical care or self-medicate. Moreover, there is a paucity of studies on the subject from developing countries and none from India. So, our study uses the drug "Promethazine" to solve both of these problems encountered by cleft surgeons, such as reducing anxiety before surgery and preventing nosocomial infection. Promethazine is considered to be safe and has been used in various other conditions like nausea/vomiting, allergic conditions, prevention of motion sickness, and pre/post-operative or obstetric sedation [18]. Razieh Fallah *et al*. evaluated the Efficacy of Chloral Hydrate and Promethazine for Sedation during Electroencephalography in Children where they a dose of 70 mg/kg chloral hydrate or promethazine 1 mg/kg orally is subjected which concluded that chloral hydrate is an effective drug in sedation and can be used for various pediatric procedure for sedation [31]. Malobika Bhattacharya *et al*. Compared the Effect of Dextromethorphan, Promethazine, and Placebo on Nocturnal Cough in Children Aged 1-12 years with Upper Respiratory Infections, and found that Nocturnal cough in URI is self-resolving and dextromethorphan and promethazine prescribed for the same are not superior to placebo [20].

Our study shows that the children taking promethazine as premedication are found to have less anxiety level before surgery and they were calm in the recovery area which reduces mental stress of the child as well as parents. Additionally, in our study, the preoperative sleep of both children and parents was recorded and they were found to sleep peacefully for 3 nights before surgery. The frequency of cough and vomiting scores were calculated and were less in children taking promethazine. Variations in heart rate were also observed and found that there was no variation in intraoperative heart rate for children taking promethazine. Research shows there is an enigma in the use of antihistamine therapy for cough in children, which is mostly unjustified [32]. Paul *et al*. showed that diphenhydramine (a first-generation H1-antagonist) and dextromethorphan were no different from placebo in reducing nocturnal cough or sleep disturbance in children [33]. But our study focuses on prophylactic dose rather than the management of cough. However there are a few limitations of our study, the US black box warning suggests promethazine is not recommended for children less than two years old which causes respiratory depression resulting in fatalities [34]. And also in some cases, promethazine has been reported to cause serious and life-threatening respiratory depression, over sedation, agitation, hallucinations, seizures, and dystonic reactions when used in children [35,36]. But our study focuses on prescribing promethazine for cleft palate children who are operated on after 2 years of age. Secondly, it is important to note that this study was conducted as a clinical trial in a specific setting and involved a limited sample size. Therefore, further research with larger sample sizes and diverse populations is necessary to validate the findings and determine the generalizability of promethazine's effectiveness in cleft palate surgeries. It is also crucial to consider individual patient characteristics, potential contraindications should be taken into consideration before prescribing promethazine to children undergoing cleft palate surgeries.

## Conclusion:

As the benefits of promethazine in cleft palate surgery rule over its adverse effects, promethazine is considered as safe as premedication for children undergoing cleft palate surgeries. Promethazine causes a reduction in Anxiety level by 34% and a 46% reduction in cold and cough, an improvement in sleep score by 38%, and the heart rate was found to be stable throughout the surgery when compared to the control group. By demonstrating the effectiveness of Promethazine in managing these issues, the study provides valuable insights for healthcare professionals and surgeons involved in the care of children with cleft palate.

## Funding:

None

## Figures and Tables

**Table 1:** represents the cough objective scale validated by Bhattacharya *et al*. [20]

A.Cough frequency score:	(1) None	(2) Occasional (<10 coughs/night; no prolonged episode)	3) Often (10-20 coughs/night; ≤2 prolonged episodes)	(4) Very often (>20 coughs/night; >2 prolonged episodes)
B.Child's sleep score:	(1) Slept all night	(2) Woke up occasionally (≤2/night)	(3) Woke up frequently (>2/night)	4) Did not sleep at all
C.Parents'sleep score:	(1) Slept all night	(2) Woke up occasionally (≤2/night)	(3) Woke up frequently (>2/night)	(4) Did not sleep at all
D.Post-tussive vomiting score:	(1) No	(2) Yes		
E.Composite symptom score:	Obtained by a cumulative score of the individual symptoms			

**Table 2:** Anxiety index

**Anxiety index**	**Group**	**N**	**Mean**	**Std deviation**	**std error of mean**	**Sig.**
	case	64	1.5	0..067	0.067	0.044
	control	64	2.89	0.893	0.112	

**Table 3:** Cough frequency score

**Cough frequency score**	**Group**	**N**	**Mean**	**Std deviation**	**Std error of mean**	**Sig.**
	case	64	1.44	0.445	0.056	0.001
	control	64	2.84	0.687	0.086	

**Table 4:** Child's sleep score

**Child's sleep score**	**Group**	**N**	**Mean**	**Std deviation**	**Std error of mean**	**Sig.**
	case	64	1.2	0.443	0.055	0.004
	control	64	2.36	0.601	0.075	

**Table 5:** Intraoperative Heart rate

	**Group**	**N**	**Mean**	**Std deviation**	**Std error of mean**	**Sig.**
Heart rate	case	64	105	7.6	0.95	0.027
	control	64	113.7	8.9	1.11	
